# Retinoic acid receptor responder 2 and lipid metabolic reprogramming: A new insight into brain metastasis

**DOI:** 10.1002/cai2.148

**Published:** 2024-10-24

**Authors:** Lulu Wang, Yan Gao

**Affiliations:** ^1^ Department of Human Anatomy, School of Basic Medical Sciences Capital Medical University Beijing China; ^2^ Beijing Key Laboratory of Cancer Invasion and Metastasis Research Beijing China

**Keywords:** brain metastases, breast cancer, lipid metabolism, retinoic acid receptor responder 2

## Abstract

The brain is a common metastatic site for carcinoma, and metabolic reprogramming is crucial for organ‐tropic metastatic formation. Li et al. found RARRES2 deficiency affected lipid metabolic reprogramming through PTEN‐mTOR‐SREBP1 pathway and promoted BCBrM. Other studies revealed that lipid metabolic reprogramming is part of metabolic adaptation to central nervous system. Overall, there is an intricate connection between lipid metabolism and brain metastases, and disrupting this connection may be a potential therapeutic target for BCBrM treatment.

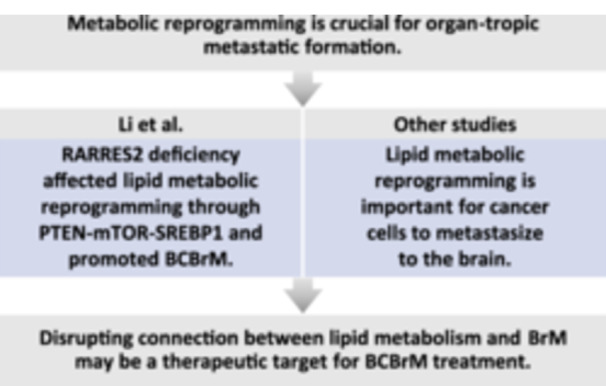

**Abbreviations**
BCBrM: breast cancer brain metastases
BrM: brain metastases
mTOR: mammalian target of rapamycin
RARRES2: retinoic acid receptor responder 2
TAG: triacylglycerol

The brain is one of the most common metastatic sites for carcinoma, especially for breast cancer, the second leading cause of brain metastases (BrM) after lung cancer [1]. During organ‐tropic metastases, cancer cells have to survive and expand in target organs through a process involving a complex interplay between invading cells and the microenvironment. While many factors govern this process, recent findings have implicated an essential role of metabolic reprogramming in determining organ‐tropic metastatic formation. The brain has a unique metabolic microenvironment in which lipids constitute ∼50% of the components. Studies have revealed the importance of fatty acid and cholesterol metabolism for the metastasis of breast cancer cells to the brain [2] and demonstrated the requirement of fatty acid synthesis in mediating HER2‐positive breast cancer cells forming metastases in the brain [3]. Li et al. further explored the underlying mechanisms of lipid metabolic reprogramming and breast cancer brain metastases (BCBrM) [4].

Li et al. performed single‐cell RNA sequencing on primary breast tumors and BCBrM samples and observed upregulated lipid synthesis and fatty acid metabolism signatures in BrM tumor cells, reinforcing previous findings [4]. To identify a potential adipokine that might be of critical importance in the initiation and development of BCBrM, the authors performed data mining and compared genes‐encoding adipokines or critical enzymes in lipid metabolism. Retinoic acid receptor responder 2 (RARRES2) was identified as the most significantly downregulated gene in BCBrM. RARRES2, also known as chemerin, is an adipokine that regulates adipogenesis and adipocyte differentiation. However, its functions in cancer cell lipid metabolism are not known. The authors demonstrated decreased expression of RARRES2 in BCBrM through public data sets and their own patient samples and identified a potential association between RARRES2 downregulation and BrM.

The authors then explored the role of RARRES2 in BCBrM formation in both in vitro and in vivo studies. RARRES2 overexpression significantly suppressed BrM following both intracranial and intracardiac injection, while RARRES2 knockdown significantly promoted breast cancer cell proliferation and invasion. Further lipidomic profiling analysis revealed increased glycerophospholipids and fatty acid synthesis levels and decreased triacylglycerols (TAGs). These changes reflected the metabolic flexibility of brain metastatic cells to use available nutrients and adapt to the metabolic environment of the brain, where it accumulates specialized lipids to support neural activity rather than relying on TAGs.

The synthesis of fatty acid and glycerophospholipid greatly burdens cellular metabolism, as large amounts of acetyl‐CoA are required, which might explain why de novo fatty acid synthesis is upregulated in brain metastatic breast cancer cells. Thus, cellular signaling pathways associated with metabolic availability and cellular energetic status, such as the mammalian target of rapamycin (mTOR) signaling, are likely to be activated. Furthermore, sterol regulatory element‐binding protein 1, a downstream regulator of mTOR, and a key transcription factor responsible for the regulation of fatty acids and TAG synthesis in lipogenic organs, is the critical factor that determines the ability of cancer cells to metastasize to the brain [3]. Li et al. observed similar findings [4]. They found activation of the mTOR‐sterol regulatory element‐binding protein 1 signaling pathway upon RARRES2 knockdown and determined that this activation may be exerted from chemokine‐like receptor 1, the principal receptor of RARRES2.

Lipid metabolic reprogramming as part of the metabolic adaptation to the central nervous system microenvironment was also previously demonstrated in acute lymphoblastic leukemia and BrM of melanoma [5, 6]. Parida et al. recently revealed that limiting mitochondrial plasticity impaired fatty acid oxidation, increased lipid droplet accumulation, and attenuated BCBrM, which suggested the therapeutic potential of targeting cellular plasticity programs to prevent metastatic recurrence [7]. Together, these findings could expand the treatment of BrM from different primary cancer types. However, some major challenges remain before clinical application. For example, inhibitors or agonists must have the ability to cross the blood–brain barrier. Additionally, metabolic plasticity may result in the activation of alternative pathways. Finally, metastatic cells may use nutrients provided by stromal cells, which may lower the efficacy of treatments targeting cancer cells.

Overall, the studies by Li and other researchers [2, 3, 4, 5, 6, 7] revealed the intricate connection between lipid metabolism and BrM. Disrupting this connection may halt BrM formation or be a potential therapeutic target. The exciting findings of these recent studies may provide new opportunities for the treatment of BCBrM.

## AUTHOR CONTRIBUTIONS

**Lulu Wang**: Conceptualization (supporting); writing—original draft (equal); writing—review and editing (lead). **Yan Gao**: Conceptualization (lead); writing—original draft (equal); writing—review and editing (supporting).

## CONFLICT OF INTEREST

The authors declare no conflict of interest.

## ETHICS STATEMENT

Not applicable.

## INFORMED CONSENT

Not applicable.

## Data Availability

Data sharing not applicable to this article as no datasets were generated or analyzed during the current study.
